# Exploring cellular diversity in lung adenocarcinoma epithelium: Advancing prognostic methods and immunotherapeutic strategies

**DOI:** 10.1111/cpr.13703

**Published:** 2024-06-30

**Authors:** Lianmin Zhang, Yanan Cui, Jie Mei, Zhenfa Zhang, Pengpeng Zhang

**Affiliations:** ^1^ Department of Lung Cancer, Tianjin Lung Cancer Center, National Clinical Research Center for Cancer, Key Laboratory of Cancer Prevention and Therapy, Tianjin's Clinical Research Center for Cancer Tianjin Medical University Cancer Institute and Hospital Tianjin China; ^2^ Department of Medical Oncology, Shanghai Pulmonary Hospital, School of Medicine Tongji University Shanghai China; ^3^ The First Clinical Medicine College Nanjing Medical University Nanjing China

## Abstract

Immunotherapy has brought significant advancements in the treatment of lung adenocarcinoma (LUAD), but identifying suitable candidates remains challenging. In this study, we investigated tumour cell heterogeneity using extensive single‐cell data and explored the impact of different tumour cell cluster abundances on immunotherapy in the POPLAR and OAK immunotherapy cohorts. Notably, we found a significant correlation between CKS1B+ tumour cell abundance and treatment response, as well as stemness potential. Leveraging marker genes from the CKS1B+ tumour cell cluster, we employed machine learning algorithms to establish a prognostic and immunotherapeutic signature (PIS) for LUAD. In multiple cohorts, PIS outperformed 144 previously published signatures in predicting LUAD prognosis. Importantly, PIS reliably predicted genomic alterations, chemotherapy sensitivity and immunotherapy responses. Immunohistochemistry validated lower expression of immune markers in the low‐PIS group, while in vitro experiments underscored the role of the key gene PSMB7 in LUAD progression. In conclusion, PIS represents a novel biomarker facilitating the selection of suitable LUAD patients for immunotherapy, ultimately improving prognosis and guiding clinical decisions.

## Graphical abstract

Developed a novel prognostic and immunotherapeutic signature for lung adenocarcinoma, revealing its effectiveness in predicting treatment outcomes and immunotherapy responsiveness, fundamentally advancing personalized cancer therapy.

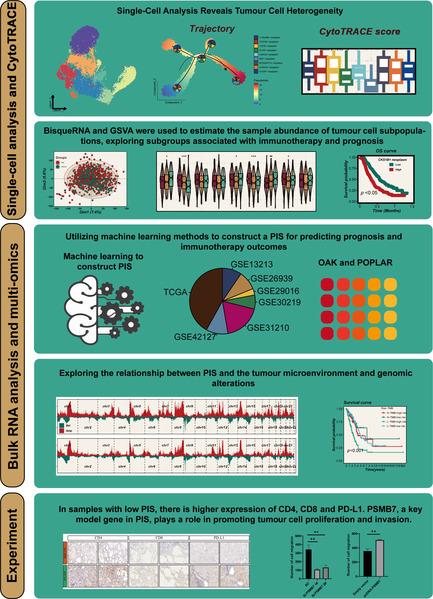

## 1 INTRODUCTION

Lung cancer (LC), a major global health issue.^1^, ^2^ predominantly occurs as non‐small cell lung cancer (NSCLC), with lung adenocarcinoma (LUAD) being its primary subtype.^3^, ^4^ Despite advancements in treatments, cure rates for advanced LUAD remain low, driving ongoing efforts to find more effective therapies and early detection methods.^5^ Immunotherapy has revolutionized cancer treatment, garnering considerable attention within the medical community. Its advantages encompass prolonged therapeutic efficacy, reduced adverse effects and applicability across a broader spectrum of malignancies.^6^ However, not all patients exhibit favourable responses to immunotherapy, posing a pivotal challenge of identifying specific cohorts amenable to its benefits. This stratification remains a paramount concern within the realm of contemporary oncology.^7^

With the continual evolution of medical technology, the landscape of LUAD research has become increasingly diversified. The advancements in single‐cell technology and bioinformatics have opened novel vistas and possibilities in comprehending the genesis, progression and therapeutic avenues concerning LUAD.^8^ Single‐cell sequencing techniques have empowered researchers to delve into LUAD investigations at cellular and molecular strata, unravelling the intricacies and heterogeneity residing within LC cells.^9^ Simultaneously, this technology enables precise identification and discrimination of diverse cellular subpopulations, unveiling specific subsets pivotal in the inception and progression of LUAD. This revelation offers pivotal clues for precise therapeutic interventions and prognostic predictions in LUAD management.^10^

In recent years, the development and application of biomarkers in LUAD research have become a focal point. Despite the identification of numerous genes and molecular markers related to the prognosis of LUAD, their effectiveness and predictive value in clinical applications remain challenging. For instance, Zhang et al. delved into the role of the basal membrane (BM) in LUAD, identifying 31 BM‐associated genes related to the prognosis of LUAD and establishing a prognostic model based on 17 key genes, accurately predicting patient outcomes.^11^ However, while these findings provide new perspectives for the prognostic prediction of LUAD, current research mainly focuses on prognostic markers, with a relative scarcity of markers for predicting the efficacy of immunotherapy. Therefore, developing markers capable of predicting the prognosis of LUAD patients and their response to immunotherapy is of significant value for selecting suitable patient groups for immunotherapy in clinical practice. This can not only assist doctors in making more accurate treatment decisions but also offer more personalized treatment options for LUAD patients, thereby improving treatment efficacy and patient quality of life.

This study endeavours to extract and meticulously analyse all LUAD cells from the NSCLC single‐cell data set curated by Salcher et al. The aim is to unravel novel cellular subpopulations within LUAD that wield significant influence on therapeutic interventions and prognostic outcomes. By scrutinizing these distinct cellular subsets, the investigation seeks to elucidate the intricate biological mechanisms underpinning their role in the treatment response and prognosis of LUAD.

## 2 METHOD

### 2.1 Data set source

The integration of 29 scRNA data sets for the analysis of the tumour microenvironment (TME) in NSCLC was undertaken by Stefan et al.^12^ Samples from primary LUAD were extracted, with a specific focus on tumour cells, to perform subclustering and further explore the heterogeneity of tumour cells. The LUAD transcriptome data and clinical data were successfully obtained from The Cancer Genome Atlas (TCGA) database (https://portal.gdc.cancer.gov), encompassing RNA sequencing data, methylation data, copy number variation data, mutation data and survival information. In addition, six data sets were obtained from the Gene Expression Omnibus (GEO) database (http://www.ncbi.nlm.nih.gov/geo) for model validation. These data sets include GSE13213^13^ (*n* = 119), GSE26939^14^ (*n* = 115), GSE29016^15^ (*n* = 39), GSE30219^16^ (*n* = 86), GSE31210^17^ (*n* = 227) and GSE42127^18^ (*n* = 134). Five hundred seventy‐seven cases of LUAD were extracted from OAK^19^ and POPLAR,^20^ 2 large clinical randomized controlled trials focusing on chemotherapy and immunotherapy for NSCLC, for the analysis in this study. The clinical information for all cohorts is comprehensively summarized in Table S1.

For the sake of ensuring uniformity and comparability of data, gene expression data underwent conversion into transcripts per million format. To mitigate potential batch effects, the ‘combat’ function within the ‘sva’ R package was applied. Furthermore, log transformation was performed on all data sets obtained from both the TCGA and GEO databases, establishing a standardized data format at the outset of the analysis.

### 2.2 The detailed steps of the single‐cell analysis process

The initial gene expression matrix underwent preprocessing utilizing the Seurat R package (version 4.2.0).^21^ Genes were required to exhibit expression in a minimum of 10 cells within each sample for inclusion. Following this, inferior cells were excluded based on specific criteria, including those with more than 5000 or fewer than 200 expressed genes, or cells with over 10% of unique molecular identifiers (UMIs) originating from the mitochondrial genome. The resultant high‐quality single‐cell transcriptome expression matrix was integrated using the harmony R package. Subsequently, a set of highly variable genes was chosen for principal component analysis (PCA), and the top 30 significant principal components were selected for Uniform Manifold Approximation and Projection dimension reduction to visualize gene expression patterns. Differentially expressed genes within each cell subpopulation were identified using the ‘FindAllMarker’ function.

### 2.3 Trajectory, CytoTRACE analysis and metabolic pathway assessment

The Monocle2 algorithm was applied to conduct developmental trajectory analysis using inferred tumour cells. The input consisted of a gene–cell matrix derived from UMI counts, scaled within the Seurat subset. A new ‘cell data set’ function was utilized to generate an object, setting the expression family parameter to negative binomial size. After dimension reduction and unit ordering, cell trajectories were inferred using default parameters. Using the CytoTRACE package^22^ to assess the stemness and differentiation potential of distinct tumour cell subpopulations. The ‘scMetabolism’ package^23^ is employed to quantify the metabolic pathway activity of distinct tumour epithelial cell subtypes.

### 2.4 Building the most valuable prognostic and immunotherapeutic signature

BisqueRNA^24^ and gene set variation analysis (GSVA)^25^ packages were used to assess the abundance of specific epithelial clusters in LUAD samples. Univariate Cox regression analysis was employed to assess the impact of key genes within specific epithelial clusters on the survival status of LUAD. Subsequently, utilizing a 10‐fold cross‐validation, we applied 101 combinations of 10 machine learning algorithms, including stepwise Cox, Lasso, Ridge, partial least squares regression for Cox (plsRcox), CoxBoost, random survival forest, generalized boosted regression modelling, elastic network, supervised principal components (SuperPC) and survival support vector machine. The aim was to identify the most valuable prognostic and immunotherapeutic signature (PIS), characterized by the highest concordance index (C‐index).

### 2.5 Mutation landscape

Genomic alterations, including recurrently amplified and deleted regions, were discerned through GISTIC 2.0 analysis. Utilizing the R package ‘maftools’,^26^ we computed the tumour mutation burden (TMB).

### 2.6 Differences in the TME

The TIMER2.0 (http://timer.comp-genomics.org/timer/) database integrates the results of multiple algorithms and summarizes the abundance of immune cell infiltration in TCGA. We used this database to observe the differences in immune cell infiltration between high and low PIS groups. In addition, we judiciously utilized the specific features of the ‘estimate’ R package^27^ to quantify immune scores, stromal scores and ESTIMATE scores for TCGA‐LUAD patients, enabling a comprehensive evaluation of the TME.

### 2.7 Clinical specimen collection and RNA sequencing

The collection of tissue samples has received ethical approval from the Medical Ethics Committee of the First Affiliated Hospital of Nanjing Medical University. These samples, categorized as AIS, MIA, or IAC by pathology experts, are obtained on the day of the surgery and are then sent to Oncocare Inc. (Suzhou, China) for RNA sequencing.

### 2.8 Immunohistochemistry

Paraffin‐embedded tissue sections were incubated for 120 min at 37°C with the primary antibodies anti‐CD8A (1:2000 dilution; Cat# ab217344; Abcam, USA), anti‐CD4 (1:500 dilution; Cat# ab133616, Abcam), PD‐L1 (1:5000 dilution; Cat# 66248‐1‐Ig; Proteintech, Wuhan, China). Following this, horseradish peroxidase‐conjugated secondary antibodies were applied and incubated for 30 minutes at the same temperature. The sections were then stained with DAB (3,3′‐diaminobenzidine) and counterstained with haematoxylin for visualization.

### 2.9 Cell lines culture

A549 LUAD cell lines were obtained from the Institute of Biochemistry and Cell Biology at the Chinese Academy of Sciences in Shanghai, China. The culture medium, containing RPMI 1640, was supplemented with 10% foetal bovine serum (FBS) and 1% antibiotics (100 U/mL penicillin and 100 mg/mL streptomycin).

### 2.10 Transfection of plasmid DNA and small‐interfering RNA

The incorporation of PSMB7 cDNA into the expression vector pcDNA3.1 was executed. Plasmid transfection was facilitated using the X‐tremeGEN™ HP DNA transfection reagent (Roche, Basel, Switzerland), whereas small‐interfering RNA (siRNA) transfection was carried out with the Lipo2000 reagent (Invitrogen, Shanghai, China), strictly following the prescribed protocols of the manufacturer. Generally, coverslips within six‐well plates were utilized for the deposition of A549 cells, and the transfection of plasmid or siRNA was performed on the subsequent day.

### 2.11 Colony formation

A quantity of 5000 cells was introduced into each well of a 6‐well plate as part of the colony formation experiment, and conventional growth medium was introduced, later substituted after 1 week. Methanol was utilized for a period of 15 min after the colonies had reached maturity within a 2‐week span, followed by staining with 0.1% crystal violet (Sigma) for 30 min. Following this procedure, the resultant clones were quantified to determine the colony‐forming capability of the clones.

### 2.12 Ethynyl deoxyuridine

Ethynyl deoxyuridine (EdU) labelling and staining processes were conducted by utilizing an EdU cell proliferation detection kit obtained from RiboBio, Guangzhou, China. After cells were introduced into 96‐well plates at a concentration of 5 × 10^3^ cells per well, a 50 μM EdU labelling medium was administered 48 h post‐transfection. The cells were subjected to a 2‐h incubation in a controlled setting at 37°C with 5% CO_2_. Subsequently, a treatment was applied to the cells using 4% paraformaldehyde and 0.5% Triton X‐100 for anti‐EdU working solution staining. Nuclei were labelled through the utilization of diamidino‐2‐phenylindole. The determination of the percentage of EdU‐positive cells was carried out via fluorescence microscopy.

### 2.13 Wound‐healing assay

Cells were placed onto a six‐well plate and cultivated until achieving a confluence range of 90%–100%. Utilizing a delicate pipette tip, cells at confluence were subjected to incision, followed by dual rinses with phosphate‐buffered saline. Microscopic images of equivalent positions in each well were recorded at 0 and 16 h utilizing a microscope (Olympus, Tokyo, Japan). The measurement of wound closure extent was assessed as a percentage of wound confluence, employing ImageJ software.

### 2.14 Invasion and migration assays

Invasion and migration assays were executed utilizing the Transwell system by Corning, which featured 24 wells with an 8 mm pore size, situated in New York, NY, USA. In the context of migration assays, a population of 5 × 10^4^ cells post‐transfection were introduced into the upper chambers of the plates, comprising 350 μL of serum‐free medium, while 700 μL of medium enriched with 10% FBS was introduced into the lower chambers. Matrigel invasion assays entailed the application of Transwell membranes pre‐coated with Matrigel (Sigma‐Aldrich). Following a 16‐h incubation, the cells residing on the upper surface were eliminated, and those that traversed the membrane to the lower surface underwent staining with methanol and 0.1% crystal violet. Photographic records were captured utilizing an inverted microscope manufactured by Olympus in Tokyo, Japan.

### 2.15 Statistical analysis

The manipulation of data, the conduct of statistical analyses and the visualization processes were carried out using the R 4.2.0 software. The estimation and comparison of subtype‐specific overall survival (OS) were performed employing the Kaplan–Meier methodology and log‐rank test. Discrepancies in continuous variables between the two groups underwent scrutiny through the implementation of the Wilcoxon test or *t*‐test. The evaluation of categorical variables included the application of the chi‐squared test or Fisher's exact test. The correction of *p*‐values was achieved by applying the false discovery rate (FDR) method. Relationships among variables were explored through the utilization of Pearson correlation analysis. All *p*‐values were computed using a two‐tailed approach, with statistical significance indicated as *p* < 0.05.

## 3 RESULTS

### 3.1 The heterogeneity of tumour cells

**FIGURE 1:**
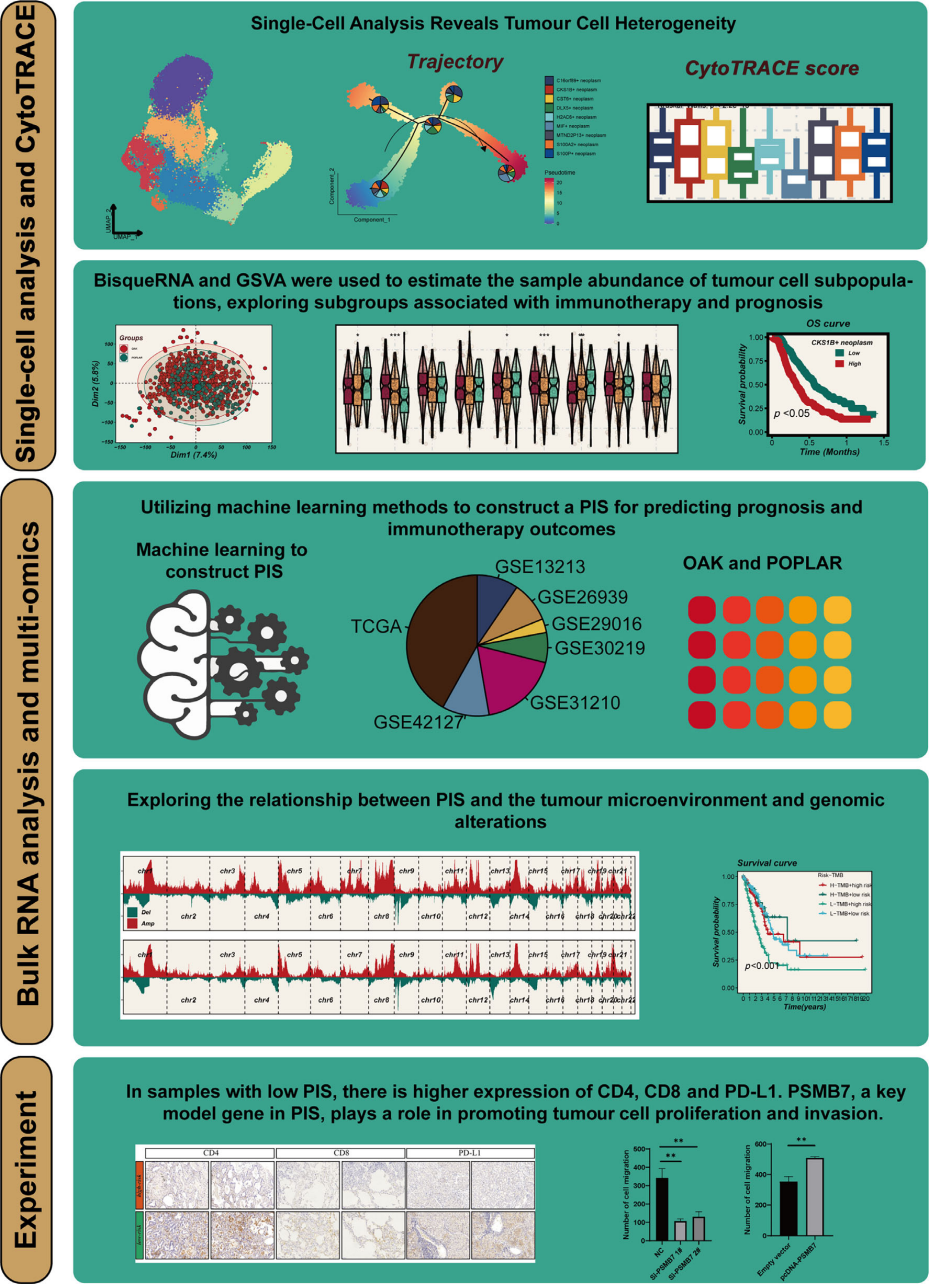
Flowchart of the analysis.

**FIGURE 2:**
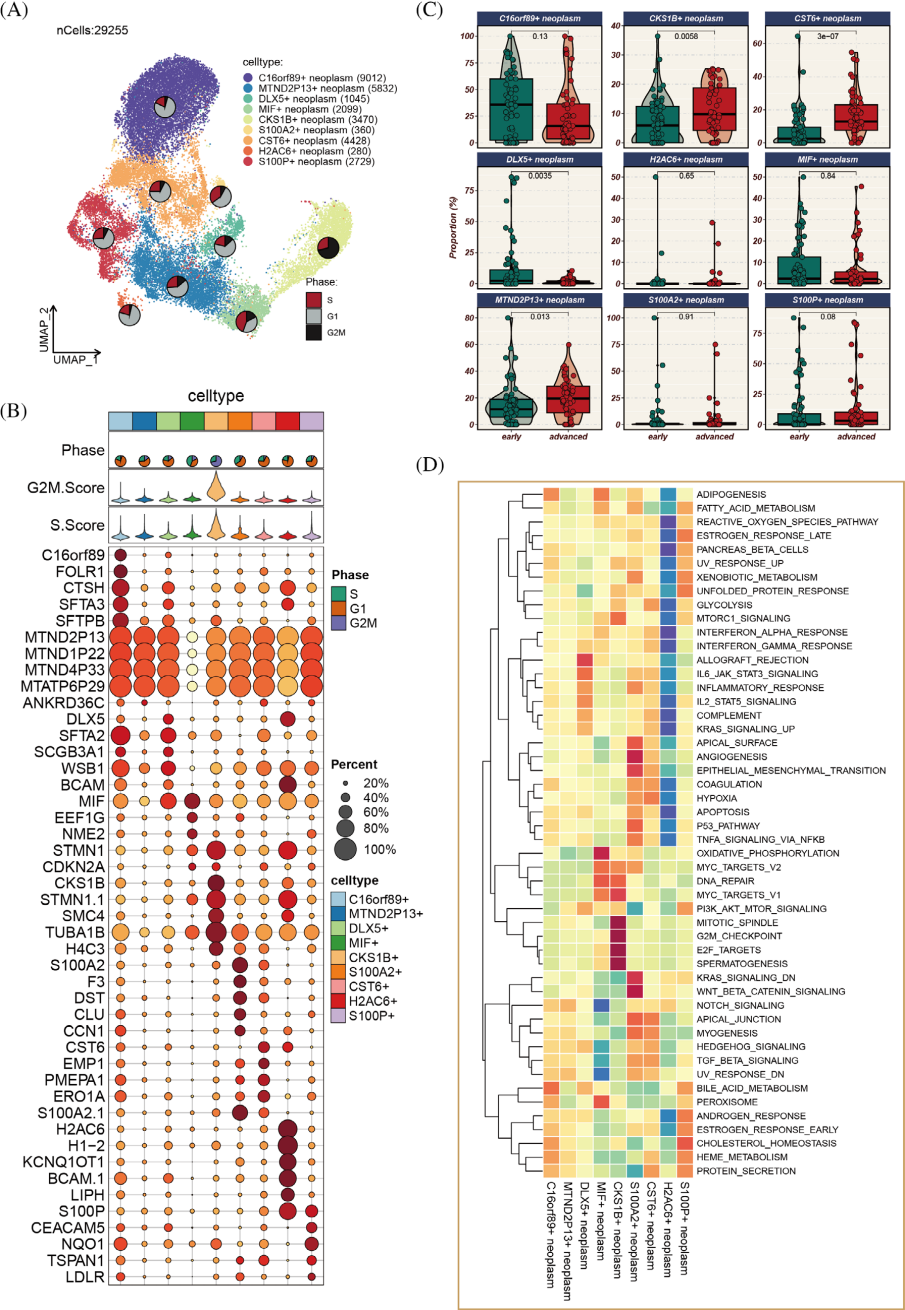
Elucidation of tumour cell heterogeneity. (A) Stratification of tumour cells into nine distinct subpopulations. (B) Identification of unique marker genes representative of each cellular subpopulation. (C) Distribution ratios of diverse tumour subgroups in the early (Stages I and II) versus late (Stages III and IV) phases of lung adenocarcinoma (LUAD), with statistical significance assessed via the Wilcoxon rank‐sum test. (D) Differential pathway enrichment across the tumour subgroups, determined through standardized analysis of hallmark gene set enrichment scores using single‐sample gene set enrichment analysis (ssGSEA).

Figure 1 shows the flow chart. Stefan et al. summarized a comprehensive analysis of scRNA‐seq data from numerous LC, extracting and clustering LUAD scRNA‐seq data into 24 primary cell types (Figure S1). Focusing on tumour cells, we further subdivided them into nine distinct groups (Figure 2A). The cell cycle states of each group are represented in pie charts. CKS1B+ cells are exclusively in the S and G2M phases, indicating high proliferative potential. Different tumour cell types exhibit diverse markers and cell cycle states (Figure 2B). CKS1B+ neoplasm, CST6+ neoplasm and MTND2P13+ neoplasm are more prevalent in advanced LUAD, while DLX5+ neoplasm is more common in early‐stage LUAD (Figure 2C). GSVA revealed variations in hallmark pathway activities among different tumour cells. Notably, CKS1B+ neoplasm significantly enriches pathways related to cell proliferation, while S100A2+ neoplasm is markedly enriched in angiogenesis and epithelial–mesenchymal transition pathways (Figure 2D).

### 3.2 Pseudo‐time and tumour stemness assessment

**FIGURE 3:**
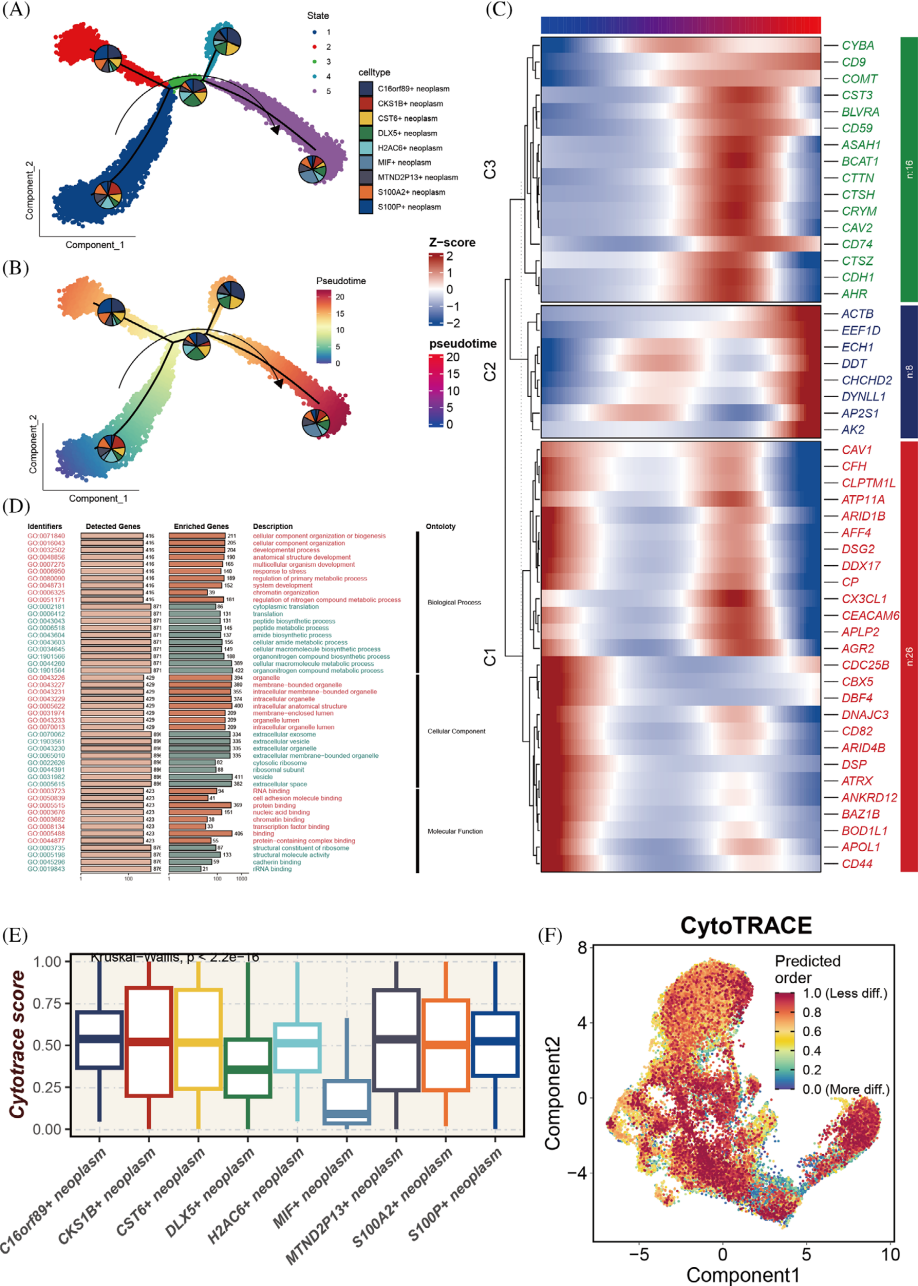
Pseudo‐time analysis and tumour stemness score assessment. (A, B) Pseudo‐time trajectory delineating the differentiation pathways of distinct tumour subgroups, with CKS1B+ tumour cells marking the initiation of development. (C) Heatmap depicting the temporal expression patterns of 50 specific genes along the pseudo‐time, categorized into three clusters (C1–C3). (D) Gene ontology (GO) enrichment analysis conducted for genes within different clusters; pathways predominantly enriched in genes from C1 are highlighted in red, whereas pathways enriched in genes from C2 and C3 are indicated in green. (E) CytoTRACE analysis employed to evaluate stemness in various tumour subgroups, with higher CytoTRACE scores indicating increased stemness; statistical significance assessed via Kruskal–Wallis test. (F) Mapping of CytoTRACE scores onto individual cells for a more intuitive representation of stemness variations across different tumour subgroups.

Pseudo‐time analysis elucidated the developmental trajectories of tumour cells (Figure 3A,B), identifying five distinct branches with varying proportions of different tumour cell types. CKS1B+ neoplasm is at the initial stage of development, gradually decreasing over time, whereas MIF+ neoplasm is at the terminal stage, increasing as time progresses. As pseudo‐time advances, the genome is divided into three clusters (C1, C2, C3), with C1 highly expressed at the start, C2 at the terminal phase and C3 in an intermediate state (Figure 3C). Enrichment analysis of genes in these three clusters (Figure 3D) indicated that Cluster 1 is primarily involved in the organization and biogenesis of intracellular structures, development of multicellular organisms, response to environmental stress, regulation of metabolic processes, chromatin structure organization and interactions between various biomolecules. CKS1B+ neoplasm, C16orf89+ neoplasm, CST6+ neoplasm, H2AC6+ neoplasm and MTND2P13+ neoplasm all demonstrate heightened tumour stemness (Figure 3E,F).

### 3.3 Immunotherapy correlation

**FIGURE 4:**
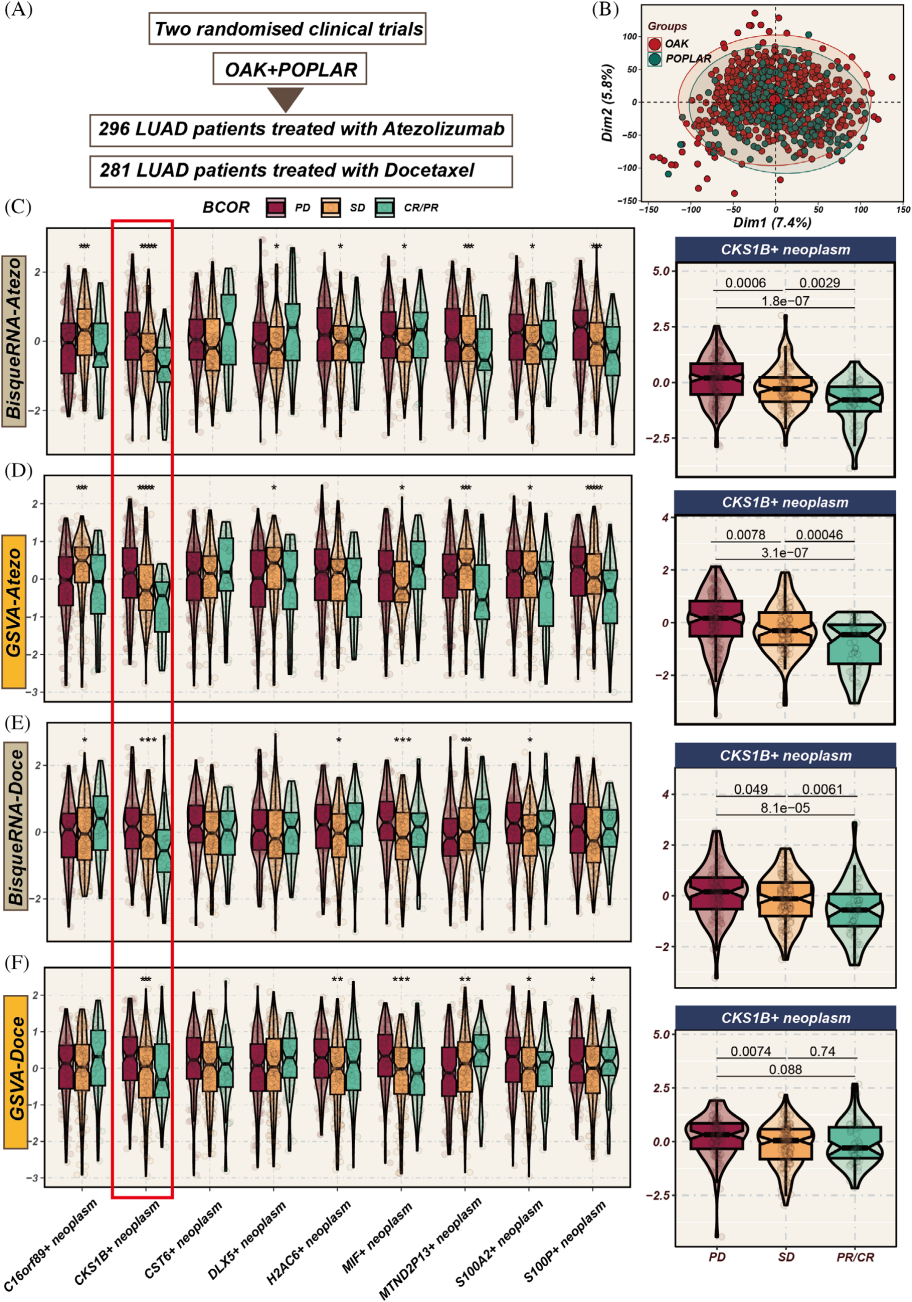
Impact of different tumour cell subgroup abundances on chemotherapy and immunotherapy efficacy. (A) Analysis includes 577 lung adenocarcinoma (LUAD) patients from OAK and POPLAR, two randomized clinical trials, receiving either chemotherapy or immunotherapy. (B) Principal component analysis (PCA) graph post‐removal of batch effects between the two cohorts. (C–F) Evaluation of the influence of varying tumour subgroup abundances on chemotherapy and immunotherapy outcomes using BisqueRNA and gene set variation analysis (GSVA) methods. Notably, the CKS1B+ tumour subgroup demonstrates significant impact in both chemotherapy and immunotherapy settings (*p* < 0.001). *p < 0.05; **p < 0.01; ***p < 0.001.

Data from 577 LUAD patients undergoing chemotherapy or immunotherapy in the OAK and POPLAR trials were analysed (Figure 4A). To integrate the data more effectively, we mitigated potential batch effects between the two cohorts (Figure 4B). Both BisqueRNA and GSVA algorithms indicated a progressive increase in the abundance of CKS1B+ tumour cell populations from partial response/complete response (PR/CR) to SD and then to PD (Figure 4C–F). There was a clear correlation between CKS1B+ neoplasm and the efficacy of immunotherapy, leading us to reasonably hypothesize that CKS1B+ neoplasm could be a potential target for LUAD immunotherapy.

### 3.4 Prognosis and metabolic heterogeneity

**FIGURE 5:**
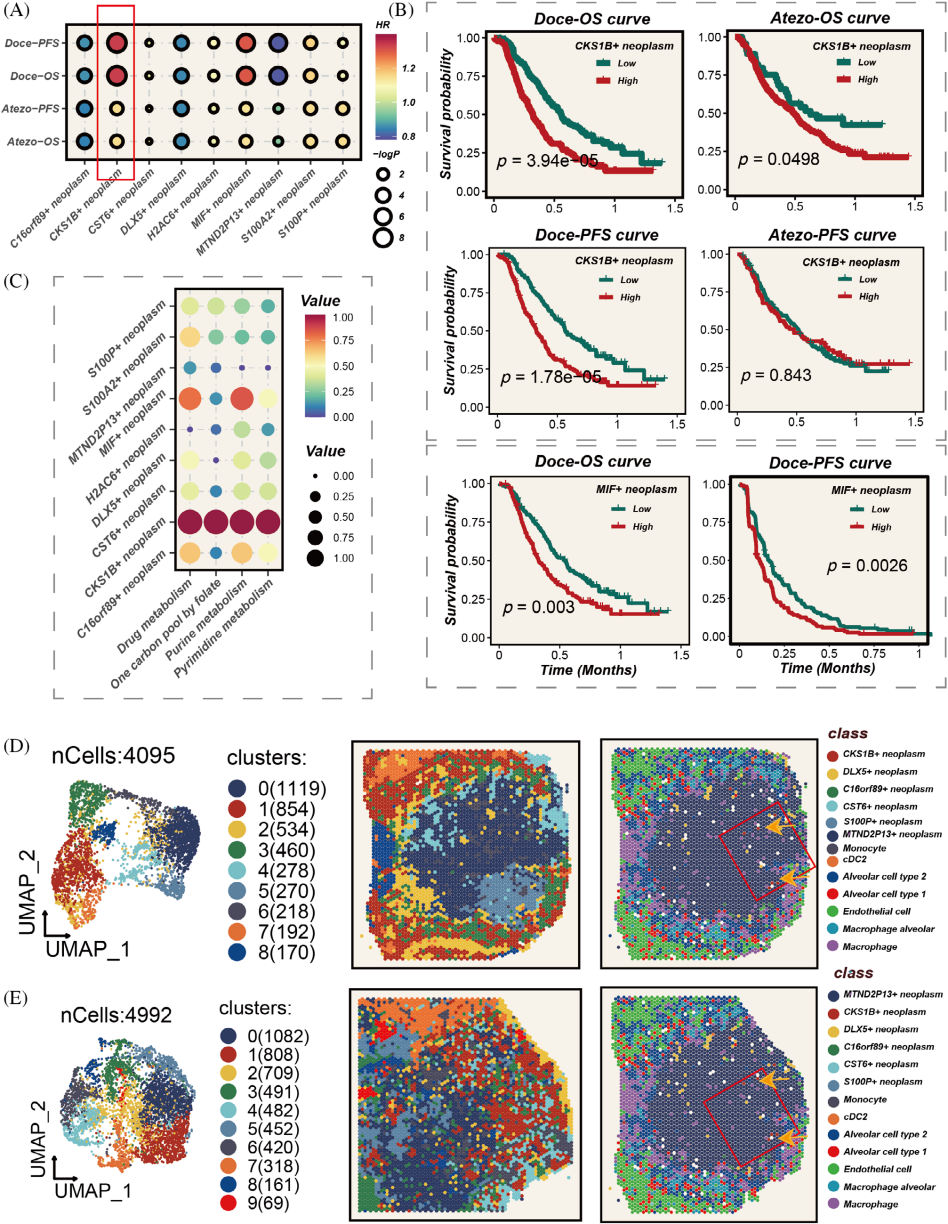
Impact of diverse tumour subgroup abundances on prognosis, and their metabolic heterogeneity and spatial localization. (A) Assessment of hazard ratios (HRs) for different tumour subgroups in chemotherapy and immunotherapy cohorts, with the CKS1B+ subgroup exhibiting HR >1, *p* < 0.05. (B) Survival differences corresponding to high and low abundances of CKS1B+ subgroup in chemotherapy and immunotherapy cohorts, with potential impact of MIF+ tumour subgroup on survival in chemotherapy cohort also observed. (D, E) Spatial localization of different tumour subgroups, highlighting the positioning of CKS1B+ tumour cells at the core of the tumour nests.

Using the ssGSEA algorithm, we assessed the abundance of tumour cell subpopulations in samples, and univariate Cox regression analysis was employed to compare prognostic significance. Regarding OS, the abundance of CKS1B+ neoplasm demonstrated strong prognostic value in both immunotherapy and chemotherapy cohorts (HR >1, *p* < 0.05, Figure 5A,B). However, its predictive capability for PFS in immunotherapy was not as pronounced. The abundance of MIF+ neoplasm showed potential predictive value for OS in the chemotherapy cohort (HR >1, *p* < 0.05). Utilizing the scMetabolism R package to analyse metabolic heterogeneity within tumour cell subgroups, we observed significant metabolic reprogramming in CKS1B+ neoplasm. This reprogramming is characterized by augmented activity in pyrimidine and purine metabolic pathways. Prior knowledge indicates that tumour cells can sometimes evade chemotherapeutic agents by modulating these nucleotide metabolism pathways, leading to chemotherapy resistance. This is corroborated by our preceding findings: the abundance of CKS1B+ neoplasm is inversely correlated with the degree of pathological remission in chemotherapy patients. This suggests that the increased nucleotide biosynthesis associated with CKS1B+ neoplasm may bolster rapid tumour cell proliferation and foster resistance to chemotherapeutic regimens. (Figure 5C), indicating its proliferative potential and possible impact on tumour progression. Focusing on CKS1B+ neoplasm, we explored its spatial localization, finding that these cells primarily concentrated in the core areas of the tumour cells (Figure 5D,E).

### 3.5 Model construction

**FIGURE 6:**
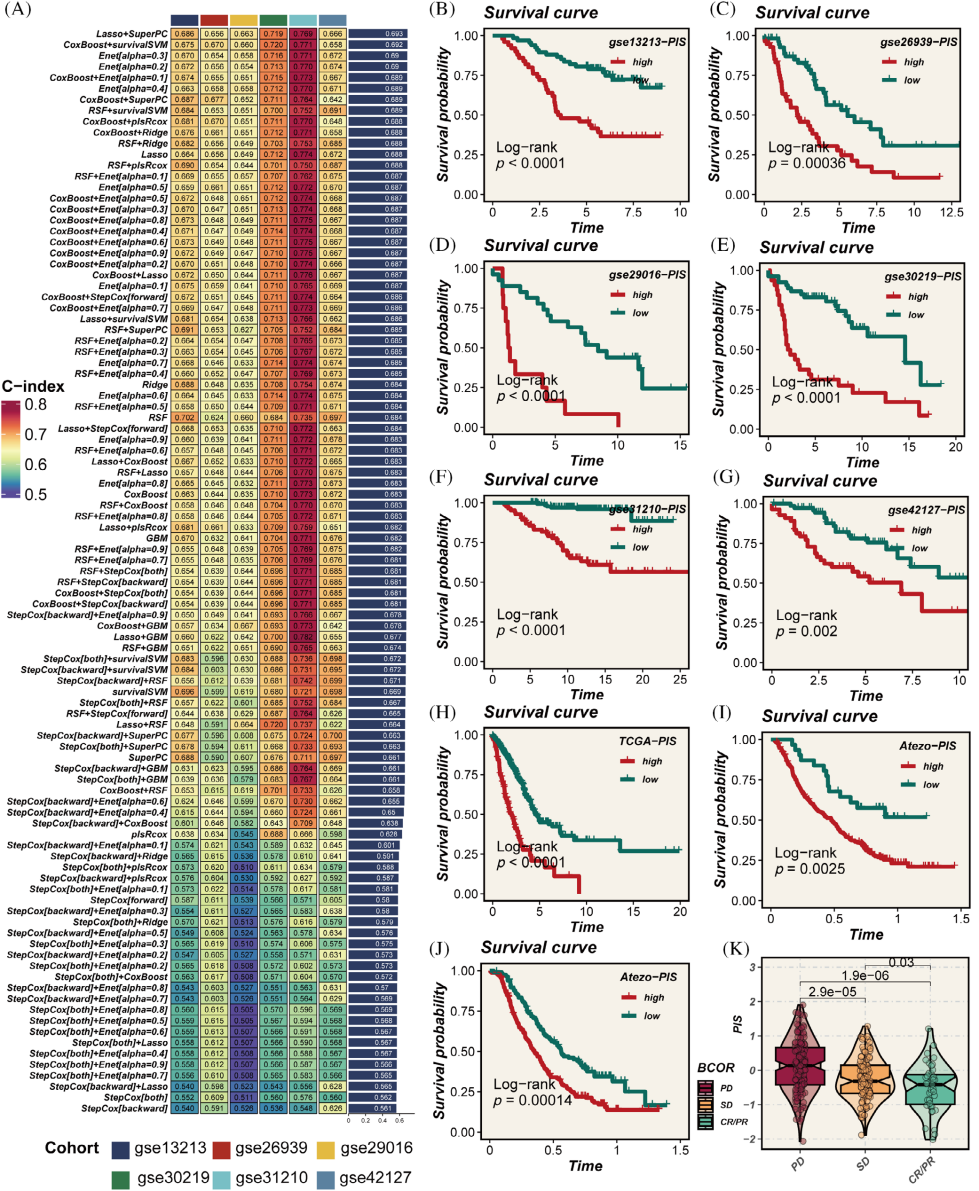
Model construction and validation. (A) Development of the prognostic immune score (PIS) using 10 machine learning methods, with the concordance index (C‐index) as the evaluation metric; Lasso + SuperPC identified as the optimal model. (B–I) Survival curves for patients with high versus low PIS across seven cohorts, with *p*‐values assessed using the log‐rank method. (J) Calculation of PIS scores in the immunotherapy cohort using the model formula, followed by evaluation of their prognostic significance. (K) Gradual increase in PIS scores across partial response/complete response (PR/CR), stable disease (SD), and progressive disease (PD) groups, indicating the score's capability to predict the efficacy of immunotherapy; *p*‐values evaluated using the Wilcoxon test.

Using marker genes of CKS1B+ neoplasm, we developed a prognostic and immunotherapy‐related signature (PIS) through a machine learning combinatorial algorithm. The TCGA data set served as the training cohort, with six GEO data sets used for validation. The C‐index average across the six validation cohorts was the criterion for model selection. Ultimately, the Lasso + SuperPC algorithm emerged as the optimal PIS (Figure 6A). The PIS distinguished patient prognosis across all seven cohorts (Figure 6B–I). Patients in the high‐PIS group exhibited worse outcomes compared with the low‐PIS group. Furthermore, we extrapolated the PIS for the immunotherapy cohort using the model's formula and found that the PIS still effectively differentiated prognosis (Figure 6J). In addition, PIS progressively increased from PR/CR to SD, then to PD (Figure 6K), indicating its potential in predicting immunotherapy outcomes.

### 3.6 Model evaluation

**FIGURE 7:**
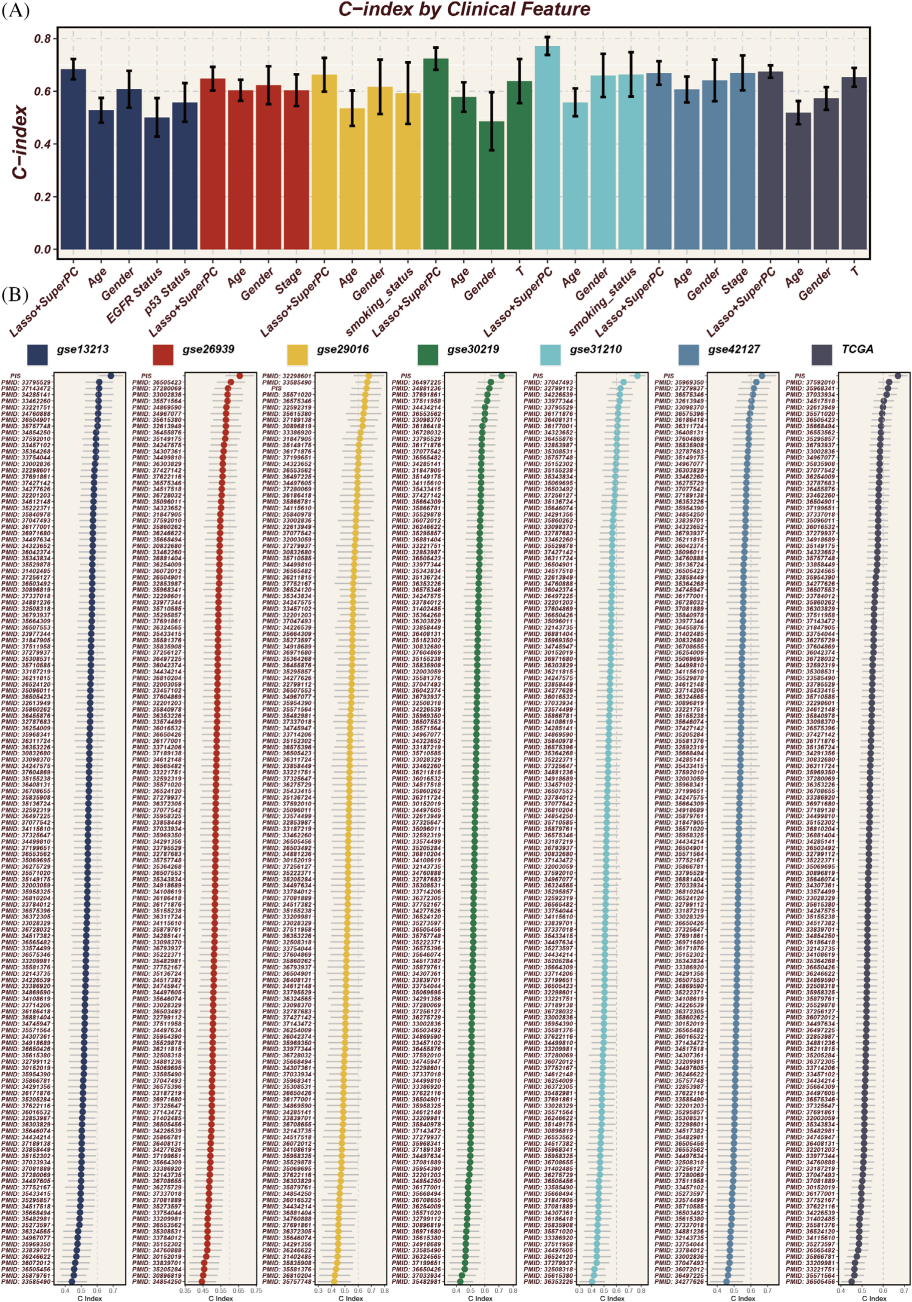
Evaluation of model performance. (A) Comparison of prognostic immune score (PIS) with other clinical features across seven cohorts, using the concordance index (C‐index) as the metric for prognostic significance assessment. (B) Comparative analysis with 144 published signatures, demonstrating PIS achieving the highest C‐index in 6 of the cohorts, underscoring its superior prognostic ability.

To assess the predictive efficacy of the PIS, we integrated clinical features from seven data sets. PIS demonstrated higher C‐index values than any other clinical feature (such as age, gender, stage, EGFR status, etc.) (Figure 7A). Subsequently, we compared PIS against 144 previously published LUAD signatures and found that PIS consistently outperformed others across 6 data sets, achieving the highest C‐index values (Figure 7B). This clearly underscores the value of PIS in predicting patient prognosis.

### 3.7 Genomic alterations

**FIGURE 8:**
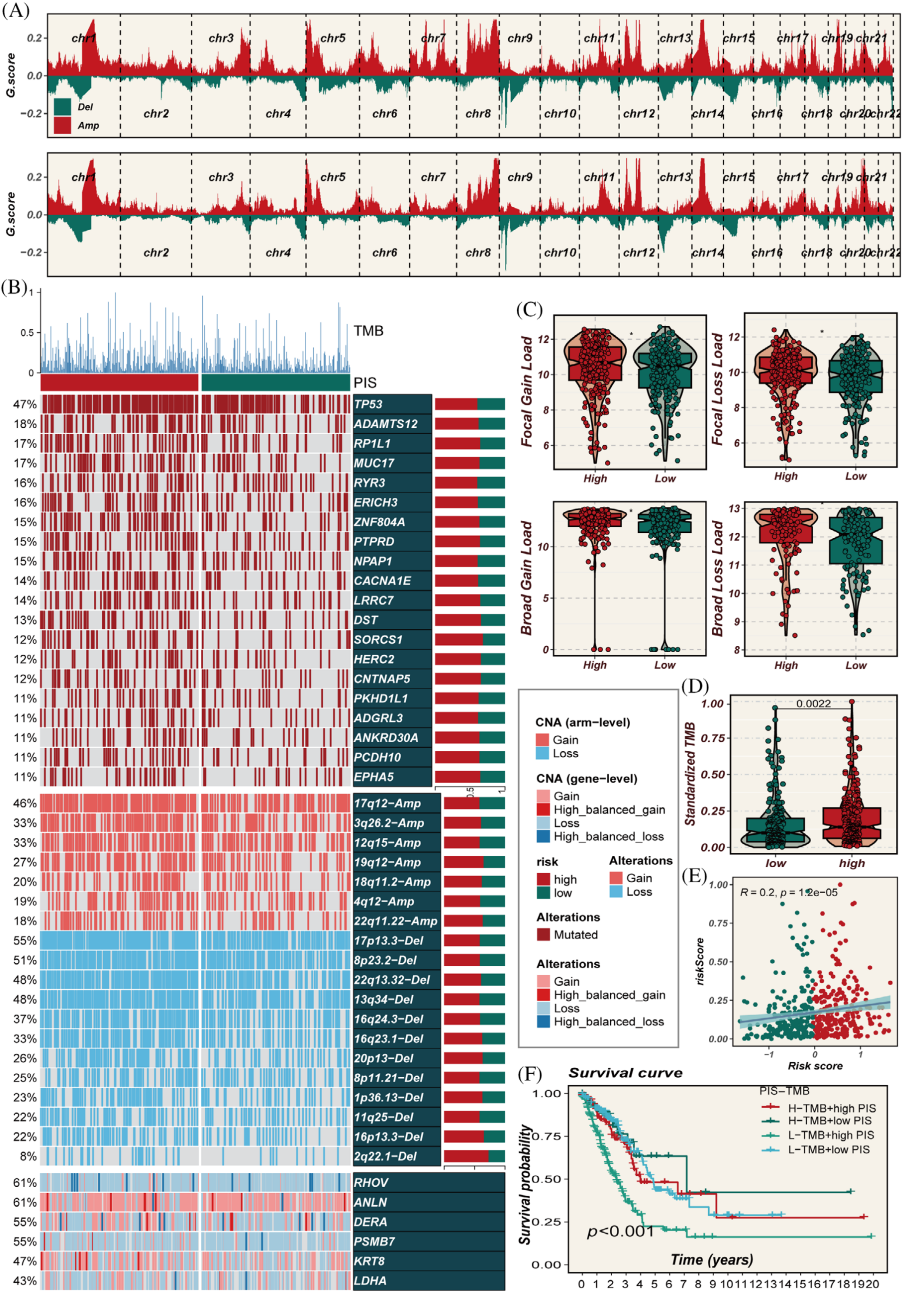
Multi‐omics characterization of prognostic immune score (PIS) in the TCGA data set. (A) Chromosomal amplifications and deletions in two PIS groups, analysed using GISTIC 2.0. (B) Genomic alterations in the high‐ and low‐PIS groups. (C) Proportions of genomic alterations in both PIS groups. (D, E) Differences in tumour mutational burden (TMB) between the two PIS groups and its correlation with PIS. (F) Survival comparison between groups classified based on median values of TMB and PIS scores, illustrating the prognostic implications of combined TMB and PIS stratification.

Figure 8A vividly shows distinct chromosomal alterations between high‐ and low‐PIS groups. The heatmap in Figure 8B highlights a markedly elevated TMB in the high‐PIS group. Analyses in Figure 8C reveal more frequent chromosomal deletions or amplifications within high‐PIS group. The high‐PIS group had a higher TMB load, and PIS was positively correlated with TMB (Figure 8D,E). Furthermore, Figure 8F indicates the worst prognosis in the L‐TMB+ high‐PIS subgroup.

### 3.8 Immune infiltration

**FIGURE 9:**
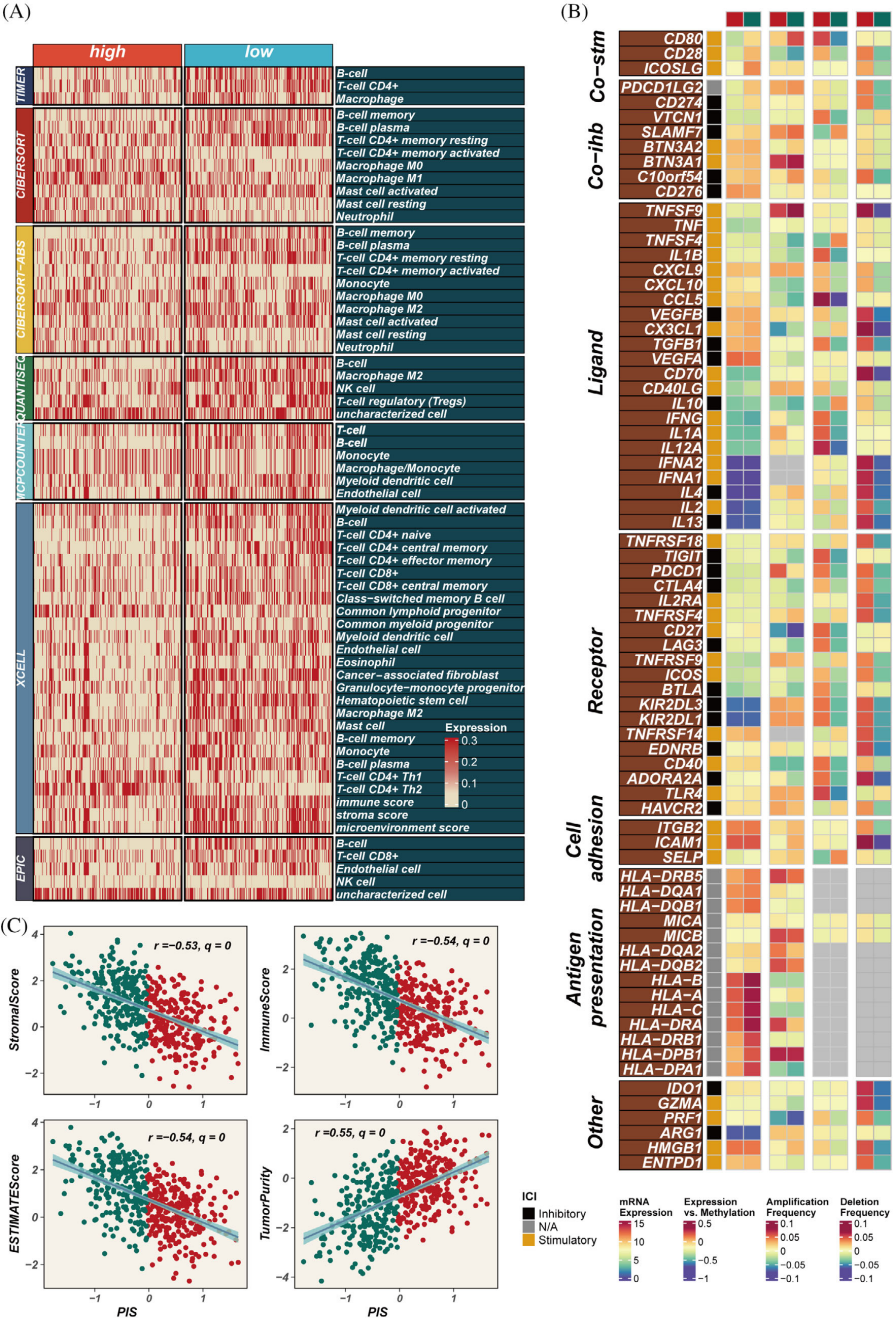
Immune infiltration assessment. (A) Comparative analysis of immune cell infiltration in groups with high versus low prognostic immune score (PIS) across seven distinct computational methods. (B) Investigation of the association between PIS scores and immune modulators. (C) Exploration of the connections among stromal scores, immune scores, ESTIMATE scores, tumour purity, as determined by the ESTIMATE package, and PIS.

To gain a deeper understanding of the immune‐related characteristics in high‐ and low‐PIS groups, we conducted a thorough investigation into their potential biological mechanisms. Results from seven algorithms indicated a higher degree of immune infiltration in the low‐PIS group, including increased infiltration of CD4 and CD8 T cells (Figure 9A). The levels of immune regulatory factors were elevated in the low‐PIS group, such as co‐stimulatory molecules CD28, CD80, ICOSLG and several antigen‐presenting related molecules like HLA‐A, HLA‐B, HLA‐C, and so forth (Figure 9B). This suggests that a lower PIS potentially facilitates immune cells' entry into the TME, enhancing their anti‐tumour activities, which might yield benefits in immunotherapy. The immune infiltration level assessed by the ESTIMATE algorithm showed that PIS negatively correlates with stromal score, immune score, ESTIMATE score and positively correlates with tumour purity (Figure 9C). The findings underscore that the low‐PIS group, with its heightened immune infiltration and regulatory factor activation, creates a TME more conducive to effective immune surveillance and response. This enhanced immune activity potentially explains the better prognosis observed in the low‐PIS group, as a robust immune presence within the tumour is often associated with improved responses to treatment and longer patient survival.

### 3.9 Pathway enrichment

**FIGURE 10:**
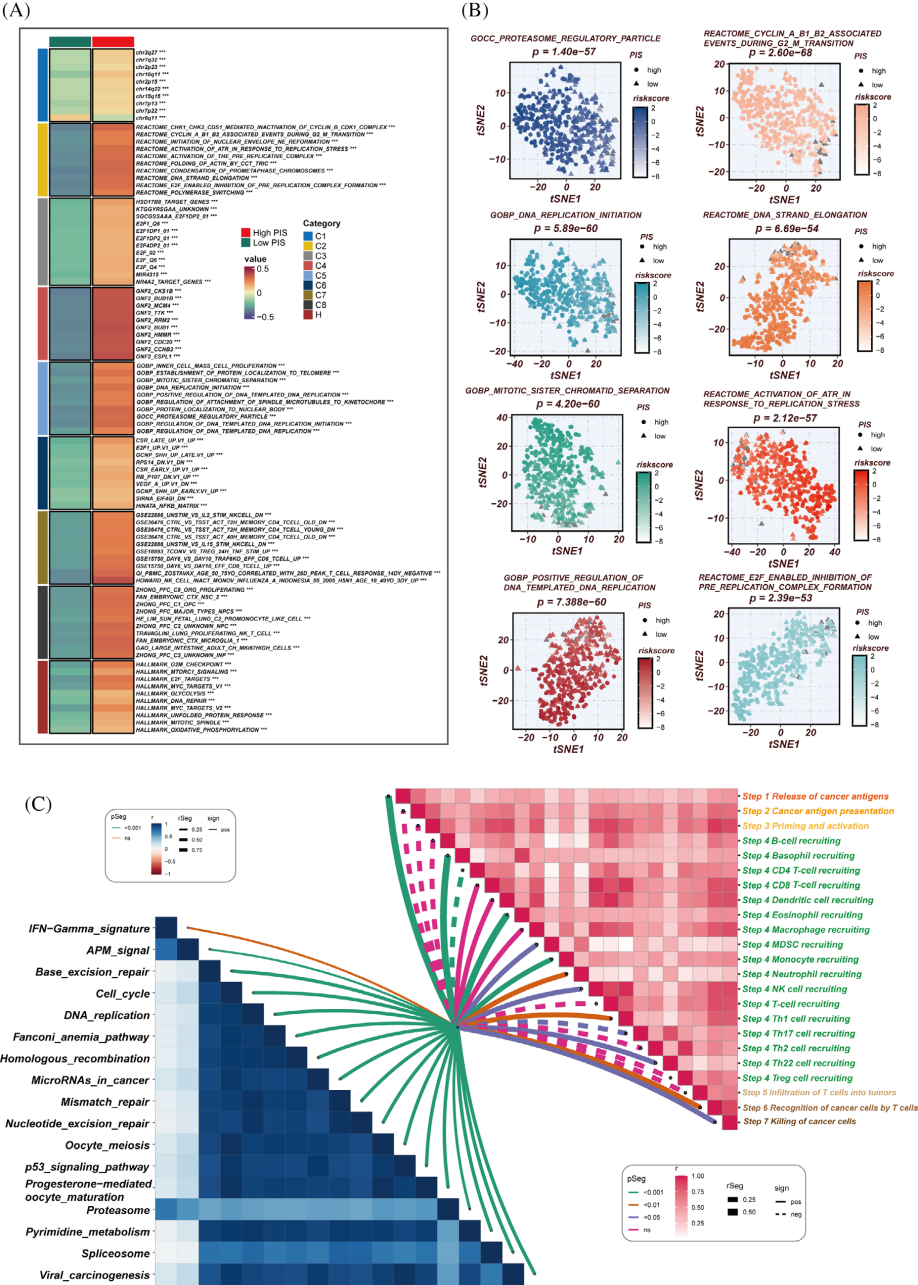
Biological characteristics of prognostic immune score (PIS) in the TCGA data set. (A) Gene set variation analysis (GSVA) based on the Molecular Signatures Database (MsigDB) delineating the biological attributes of the two PIS groups. (B) T‐distributed stochastic neighbour embedding (T‐SNE) plots of gene ontology (GO) and Kyoto Encyclopaedia of Genes and Genomes (KEGG) pathways, highlighting the differences in pathway activities between the high‐ and low‐PIS groups. (C) Evaluation of the correlation between cancer immune cycle, immunotherapy pathways, and PIS using GSVA.

The primary pathways enriched in the high‐PIS group include cell cycle regulation, DNA replication and repair, chromosomal structure alterations and protein localization and function, all of which are closely associated with tumour progression (Figure 10A,B). The aberrant activation or suppression of these pathways leads to uncontrolled cell cycle progression, increased DNA replication stress, accumulation of genetic variations and significant chromosomal structural changes. These collectively promote limitless proliferation of tumour cells, genetic instability and enhanced invasiveness and metastatic potential. Such complex molecular and cellular mechanism alterations drive rapid tumour progression and deterioration, indicating higher disease risk and poorer prognosis. PIS shows significant positive correlations with various signalling pathways and the cancer‐immunity cycle (Figure 10C). The low‐PIS group, in contrast, exhibits a favourable prognosis, likely due to a less aggressive tumour biology. This group is characterized by a lower degree of enrichment in pathways associated with uncontrolled proliferation and genetic instability. Furthermore, the enhanced immune infiltration in the low‐PIS group suggests a more active immune surveillance and response within the TME. This heightened immune presence may lead to better control over tumour growth and spread, translating to improved responses to treatments and longer patient survival. The low‐PIS group's TME, therefore, may be less conducive to tumour progression and more responsive to therapeutic interventions, contributing to the observed better prognosis.

### 3.10 Experimental validation

**FIGURE 11:**
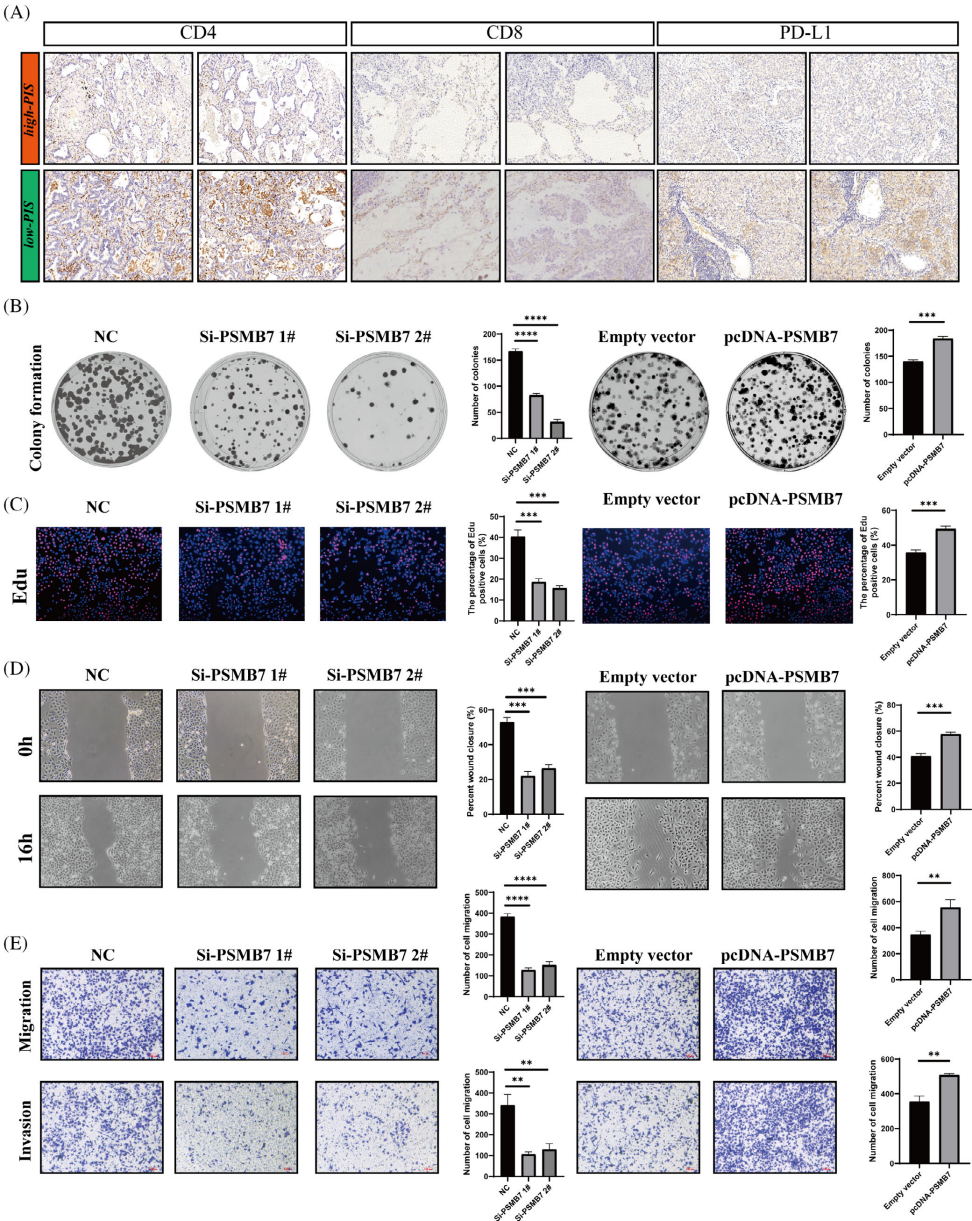
The influence of PSMB7 on clonogenicity, proliferation, and migration in lung adenocarcinoma (LUAD) cells. (A) Tissue section analysis to validate the expression differences of CD8A, CD4, and PD‐L1 between high‐ and low‐prognostic immune score (PIS) groups. (B) Colony formation assay to assess the clonogenic capability of A549 cells. (C) Ethynyl deoxyuridine (EdU) incorporation assay to analyse the proliferation of A549 cells post‐transfection. (D) Wound healing assay to evaluate the impact of si‐PSMB7 and pcDNA‐PSMB7 transfection on the migratory ability of A549 cells. (E) Transwell assay to examine the migration and invasion capacities of transfected A549 cells. **p < 0.01;***p < 0.001;****p < 0.0001.

Previous studies have shown that CD8A and CD4 are associated with the presence of CD8+ T cells and CD4+ T cells, respectively, both of which can exert anti‐tumour effects and indicate a better immune therapy response. Meanwhile, PD‐L1, an immune checkpoint protein, when highly expressed, may predict better efficacy of immunotherapy. We collected 14 LUAD samples for transcriptome sequencing and calculated their PIS scores. The top two samples with the highest PIS scores were categorized into the high‐PIS group and the two samples with the lowest scores into the low‐PIS group. Immunohistochemistry confirmed higher expression of CD8A, CD4 and PD‐L1 in the low‐PIS group compared to the high‐PIS group (Figure 11A). PIS is constituted by multiple model genes, among which PSMB7 showed a significant positive correlation with PIS (correlation = 0.69, Figure S2A). To explore the functional role of PSMB7 in LUAD tumorigenesis, we manipulated the expression of PSMB7 in A549 cells using specific siRNA and an overexpression plasmid (Figure S2B). Colony formation and EdU assays were conducted to determine the role of PSMB7 in cell proliferation. Colony formation assays indicated that PSMB7 expression impacted clonogenic ability (Figure 11B), and EdU assays revealed a significant effect of PSMB7 on LUAD cell proliferation (Figure 11C). Next, we tested the potential impact of PSMB7 on cell migration and invasion. Wound healing assays showed that the knockdown of PSMB7 significantly impeded wound closure (Figure 11D). Transwell assays also demonstrated that PSMB7 knockdown inhibited tumour migration. Conversely, the overexpression of PSMB7 significantly promoted cell migration (Figure 11E). Furthermore, the knockdown of PSMB7 restrained LUAD cells from invading through matrices, whereas the overexpression of PSMB7 facilitated their invasion (Figure 11E).

## 4 DISCUSSION

As a groundbreaking approach in cancer therapy, immunotherapy functions by activating or bolstering the patient's immune system to identify and eradicate cancer cells while mitigating severe damage to normal cells.^28^ Immunotherapy has found extensive application in treating various cancers such as renal cell carcinoma, endometrial carcinoma, LC, nasopharyngeal carcinoma, among others, yielding notable efficacy and generally presenting fewer side effects.^29^, ^30^, ^31^, ^32^ However, clinical observations indicate that only a subset of patients exhibit a response to immunotherapy. Presently, scientists speculate this phenomenon may relate to tumour type, the immune status of the patient and other factors, yet the precise biological mechanisms remain elusive.^33^, ^34^ Single‐cell sequencing technology holds unique advantages in unravelling tumour cell heterogeneity, identifying therapeutic targets and advancing personalized treatments, as corroborated by numerous past studies.^35^, ^36^

In our investigation, we meticulously analysed 29,255 tumour cells from LUAD, drawing on single‐cell data compiled by Stefan et al. We pinpointed a distinct cell subpopulation (characterized by CKS1B+ neoplasm) that demonstrates a significant correlation with the response to chemotherapy and immunotherapy in LUAD. Notably, this subgroup is distinguished by an enhanced stemness characteristic and is positioned at an early stage of pseudo‐time development, suggesting a foundational role in tumour genesis and evolution. The development and progression of malignant tumours are closely linked to dysregulation within the cell cycle and deviant cellular differentiation processes. The CKS1B gene, in this context, encodes a critical regulatory subunit that plays a pivotal role in modulating the cell cycle, underscoring its potential significance in cancer biology and therapy.^37^ Studies suggest that heightened expression of CKS1B may be correlated with abnormal proliferation, malignant transformation, invasiveness and prognosis of tumour cells.^38^, ^39^, ^40^

Li et al. found that CKS1B is overexpressed in pancreatic tumours, correlating with increased immune infiltration and cancer cell invasiveness.^41^ Subsequently, utilizing machine learning techniques, we constructed the PIS model, comprising marker genes of CKS1B+ tumours (RHOV, ANLN, DEAR, PSMB7, KRT8, LDHA). This model was validated across seven independent cohorts, consistently demonstrating exceptional predictive accuracy. Further analysis of TMB, immune infiltration and drug sensitivity underscored the robust discriminatory power of PIS. Ultimately, immunohistochemistry was employed to validate the differential immune microenvironments between high‐ and low‐PIS groups. The low‐PIS group appeared more capable of recruiting CD4 or CD8 T cells into the TME to kill tumour cells.

In addition, we conducted in vitro experiments to validate one of the characteristic genes, PSMB7. The results revealed a marked reduction in the invasiveness and migratory potential of LUAD cells upon the knockdown of the PSMB7 gene. Literature review indicates that the protein encoded by the PSMB7 gene serves as a subunit of the proteasome, engaging in intracellular protein degradation processes, including the regulation of cell cycle, protein homeostasis and antigen presentation.^42^ Studies suggest that the overexpression of PSMB7 in certain tumours might be associated with tumour proliferation, invasion and metastasis. Research links PSMB7 overexpression to tumour progression, drug resistance in multiple myeloma and shorter survival in breast cancer, underscoring its potential as a marker for cancer severity and treatment response.^43^, ^44^ In our study, we found a notable positive correlation between PSMB7 and PIS scores, in vitro experiments also confirmed the critical role of the key gene PSMB7 in enhancing the invasive and migratory potential of LUAD.

Certainly, this study has some limitations. First, it is a retrospective study based on public databases. Second, the sample size in the validation data set is limited, necessitating further independent validation. Third, the immunotherapy cohorts used in this study, OAK and POPLAR, have limitations in their original clinical characteristic data; histological types are only categorized as non‐squamous and squamous. Consequently, we were compelled to treat the non‐squamous data as indicative of LUAD for the purposes of our analysis. Lastly, due to experimental constraints, knockdown experiments were not performed on the other five genes, and additional in vitro evidence is lacking.

In summary, we have developed a novel PIS capable of accurately identifying subgroups of LUAD patients suitable for immunotherapy, providing a valuable tool for the precision treatment of LUAD. Furthermore, our findings confirm that PSMB7 may be a potential therapeutic target for the progression of LUAD.

## AUTHOR CONTRIBUTIONS

The study was conceived and designed by P.Z. Data collection was conducted by YC. JM performed the statistical analysis. The first draft of the manuscript was written by PZ. The experiment was performed by PZ. The final approval of the submitted version was given by LZ and ZZ. All authors contributed to the manuscript and approved the submitted version.

## FUNDING INFORMATION

This work was supported by the Tianjin Natural Science Foundation under Grant/Award Number 21JCYBJC01020.

## CONFLICT OF INTEREST STATEMENT

It is hereby declared by the authors that the research was carried out without the presence of any potential conflict of interest arising from commercial or financial relationships.

## Supporting information


**Figure S1.** Stefan et al. conducted an extensive analysis of single‐cell RNA sequencing (scRNA‐seq) data from various lung cancers, categorizing LUAD scRNA‐seq data into 24 distinct primary cell types.

**Figure S2.** (A) The correlation between PSMB7 and PIS. (B) Specific siRNA and overexpression plasmids were used to regulate the expression of PSMB7 in A549 cells.

**Table S1.** Summary of clinical characteristics across lung adenocarcinoma cohorts.

**Data S1.** The expression profiles of the lung adenocarcinoma cohorts.

## Data Availability

All datasets pertinent to this study are accessible through the TCGA database (http://cancergenome.nih.gov/), GEO database (https://www.ncbi.nlm.nih.gov/geo/), or the data availability sections of the relevant publications. All data relevant to this investigation, whether generated or analyzed, are comprehensively detailed in this manuscript and its supplementary materials. For further inquiries or data requests, interested parties are advised to reach out to the corresponding authors.
