# Supplementary figures and images for: Exploring cellular diversity in lung adenocarcinoma epithelium: Advancing prognostic methods and immunotherapeutic strategies
Source: Cell Prolif. 2024 Jun 30;57(11):e13703. doi: 10.1111/cpr.13703 (PMC11533061; doi:10.1111/cpr.13703)

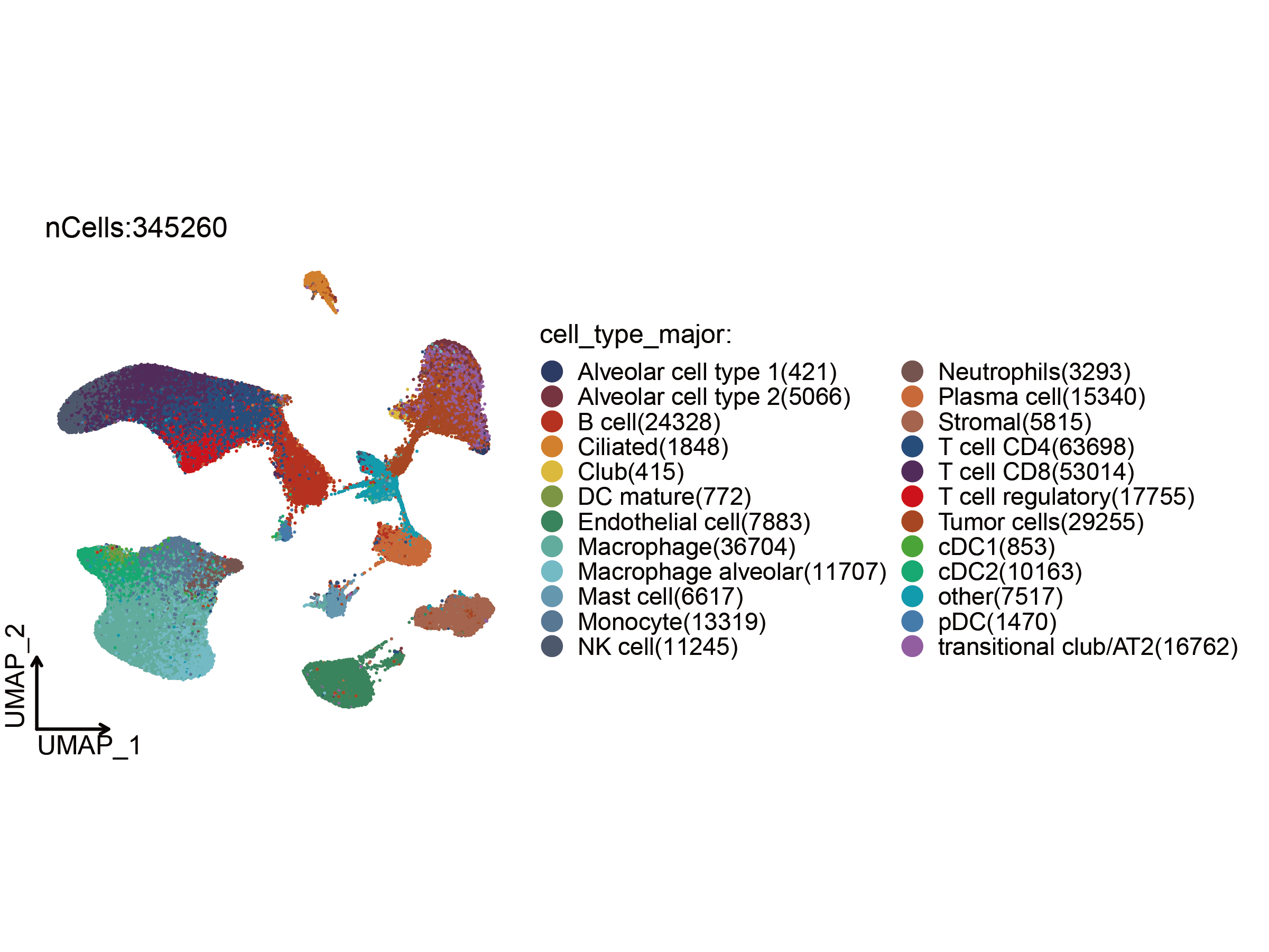

Supplement: Supplementary file 1 — Figure S1. Stefan et al. conducted an extensive analysis of single‐cell RNA sequencing (scRNA‐seq) data from various lung cancers, categorizing LUAD scRNA‐seq data into 24 distinct primary cell types. [file CPR-57-e13703-s004.tif]

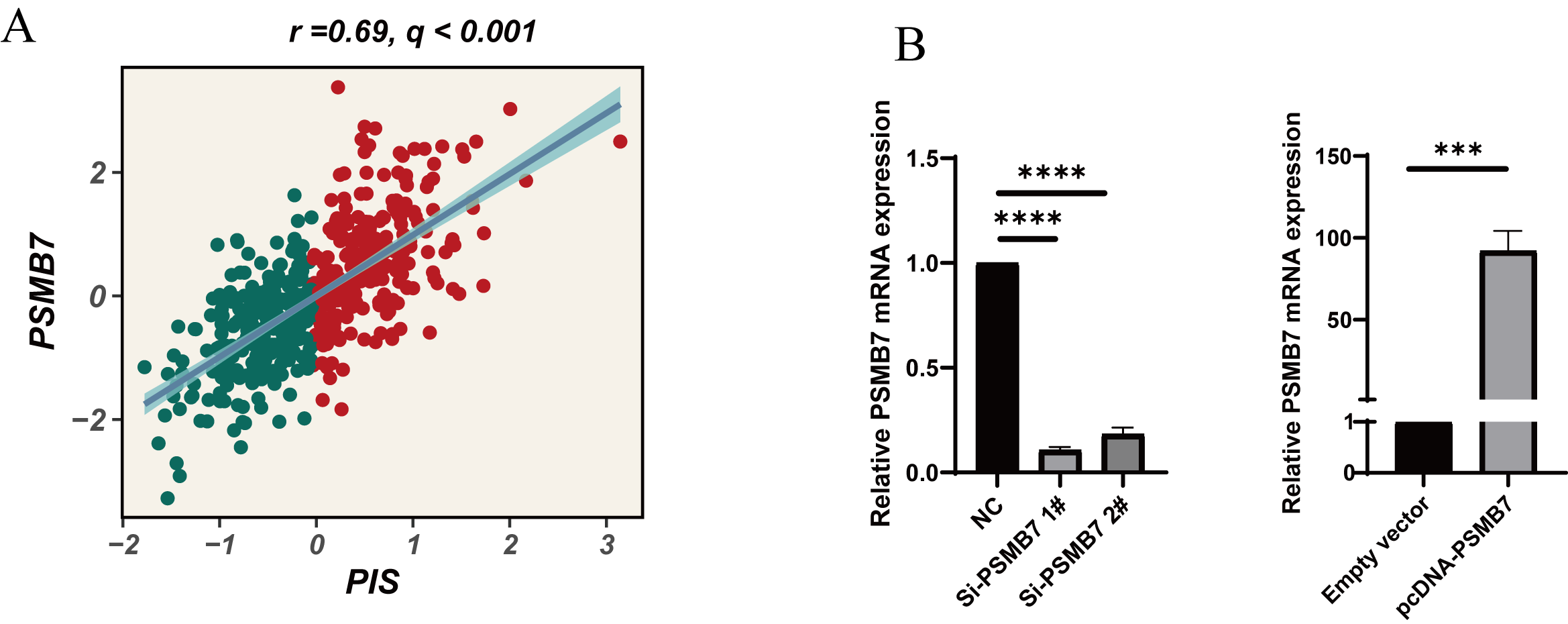

Supplement: Supplementary file 2 — Figure S2. (A) The correlation between PSMB7 and PIS. (B) Specific siRNA and overexpression plasmids were used to regulate the expression of PSMB7 in A549 cells. [file CPR-57-e13703-s001.tif]
